# Reliable Stenosis Detection Based on Thrill Waveform Analysis Using Non-Contact Arteriovenous Fistula Imaging

**DOI:** 10.3390/s24155068

**Published:** 2024-08-05

**Authors:** Rumi Iwai, Takunori Shimazaki, Jaakko Hyry, Yoshifumi Kawakubo, Masashi Fukuhara, Hiroki Aono, Shingo Ata, Takeshi Yokoyama, Daisuke Anzai

**Affiliations:** 1 Department of Dental Anesthesiology, Kyushu University Hospital, Fukuoka 819-0395, Japan; iwai.rumi.650@s.kyushu-u.ac.jp; 2 Department of Clinical Engineering, Faculty of Health Care, Jikei University of Health Care Sciences, Osaka 532-0003, Japan; t-shimazaki@juhs.ac.jp (T.S.); y-kawakubo@juhs.ac.jp (Y.K.); 3 Graduate School of Engineering, Osaka Metropolitan University, Osaka 558-8585, Japan; ata@omu.ac.jp; 4 Graduate School of Engineering, Nagoya Institute of Technology, Nagoya 466-8555, Japan; hyry.jaakko@nitech.ac.jp; 5 Department of Clinical Engineering, Shikoku Central Hospital of the Mutual Aid Association of Public School Teachers, Ehime 799-0193, Japan; 6 Department of Dental Anesthesiology, Faculty of Dental Science, Kyushu University, Fukuoka 812-8582, Japan; yokoyama@dent.kyushu-u.ac.jp

**Keywords:** arteriovenous fistula, thrill waveforms, stenosis, hemodialysis, non-contact imaging

## Abstract

Hemodialysis therapy is an extracorporeal circulation treatment that serves as a substitute for renal function. In Japan, patients receive this efficient four-hour treatment, three times per week, allowing them to maintain a social life nearly equivalent to that of healthy individuals. Before the treatment, two punctures are performed to establish extracorporeal circulation, and a high blood flow rate is essential to ensure efficient therapy. Specialized blood vessels created through arteriovenous fistula (AVF) surgery are utilized to achieve high blood flow rates. Although the AVF allows safe and efficient dialysis treatment, AVF stenosis leads to a serious problem in dialysis. To early detect this abnormal blood flow, auscultation and palpation methods are widely used in hospitals. However, these methods can only provide qualitative judgment of the AVF condition, so the results cannot be shared among other doctors and staff. Additionally, since the conventional methods require contact with the skin, some issues require consideration regarding infection and low reproducibility. In our previous study, we proposed an alternative method for auscultation using non-contact optical imaging technology. This study aims to construct a reliable AVF stenosis detection method using Thrill waveform analysis based on the developed non-contact device to solve the problem with the contact palpation method. This paper demonstrates the performance validation of the non-contact imaging in the normal AVF group (206 total data, 75 patients, mean age: 69.1 years) and in the treatable stenosis group (107 total data, 17 patients, mean age: 70.1 years). The experimental results of the Mann–Whitney U test showed a significant difference (p=0.0002) between the normal and abnormal groups, which indicated the effectiveness of the proposed method as a new possible alternative to palpation.

## 1. Introduction

Approximately 4 million patients receive kidney replacement therapy (KRT) all over the world, of which 69% are hemodialysis patients [1]. Hemodialysis treatment removes waste products, such as the final breakdown products of dietary proteins and metabolic products from muscle metabolism. It also corrects electrolytes in the blood and removes excess water. The principle in hemodialysis is diffusion and its efficiency is important. A filter called a dialyzer is used, which consists of 10,000 hollow fibers, the sides having countless holes of a nanometer size. Diffusion occurs through the hollow fibers when blood is passed inside these fibers and passing dialysate occurs in the opposite direction on the outside of the fiber. A stable and high blood flow rate is essential for improving the diffusion efficiency.

Because hemodialysis requires extracorporeal circulation at high blood flow rates, an arteriovenous fistula (AVF) is mainly used in Japan, which is an anastomosis between the radial artery (hereafter referred to as the artery) and the cephalic vein (hereafter referred to as the vein) [2,3,4,5,6,7]. This allows blood to flow from arteries with high internal pressure to veins with low internal pressure, causing veins to become distended and allowing high flow rates to be easily obtained from shallow veins. This enables stable dialysis therapy; however, stenosis of the AVF remains a significant issue due to factors such as venous tortuosity, vascular wall thickening, the presence of venous valves, blood pressure drops during treatment and thrombosis [8,9,10,11]. When stenosis becomes challenging to treat, invasive procedures such as percutaneous transluminal angioplasty (PTA) or venous stenting are performed [12,13,14]. Stenosis not only reduces dialysis efficiency, but also imposes substantial physical and mental burdens on patients as it worsens, significantly decreasing their quality of life (QoL).

Because AVFs are easily stenotic vessels, blood flow is checked by auscultation and palpation before and after treatment. The auscultation is a method of listening to the pulsating and turbulent sounds produced at the anastomosis with a stethoscope, and palpation is a method of palpating the blood vessel by feeling the vibrations of the blood vessels (called Thrill) produced by the pulsating and turbulent flow at the anastomosis from the skin surface. The difference between a normal and stenotic AVF is that, when auscultating a normal AVF, there is a laminar blood flow, i.e., a constant or regular flow—you can hear the alternating strong and weak sounds, as well as during palpating, you can also feel the finer vibrations. However, when auscultating a stenotic AVF, this laminar flow changes into a turbulent one, where the high-frequency sounds of fistula bruit, i.e., a vascular murmur, increase, and during palpating the Thrill becomes smaller.

Both methods are simple and reliable screening methods, but they only allow qualitative judgment and data cannot be shared among others. In addition, contact infection must be taken into consideration because of skin contact, and reproducibility is low because the method varies greatly depending on the way the stethoscope or fingers are applied. Once stenosis is detected, the entire arm is coated with gel, and an ultrasound examination is conducted to identify the location of the stenosis [15,16,17]. This procedure requires skilled technicians and is time-consuming. Therefore, there is a pressing need to address the challenges of existing methods and to achieve early detection of stenosis.

In existing research, there are studies that quantify blood flow using wavelet transforms on sounds acquired from small microphones [18], determine stenosis [19], and assess severe stenosis using the Burg AR model [20]. Various analytical methods using multivariate Gaussian distribution (MGD) models have also been proposed [21]. Furthermore, analyses of blood flow conditions using photoplethysmography and neural networks [22], as well as the classification of stenosis levels, have been conducted [23]. As for non-contact methods, there are approaches for detecting stenosis sites using a CCD camera to capture images from above, and the blood flow is estimated by the slight movements of the AVF by using a digital camera that captures the images from the side.

In our previous research, we proposed a method using a light source and camera to address the limitations of existing techniques and quantify the AVF waveform amplitude, offering an alternative to the stethoscope [24]. This approach allows for the acquisition of comprehensive AVF information from the entire image at once, enabling the detection of stenosis without the need for probing. We also investigated how to determine if a whole arm is either normal or has stenosis (two-class classification) by using a machine learning algorithm, such as the Gradient-Boosting Decision Tree [25]. In this study, we further investigated the frequency bands of Thrill using the same method and validated the effectiveness of stenosis detection based on quantified values.

## 2. Principle Theory

### 2.1. Thrill Mechanism

**Figure 1:**
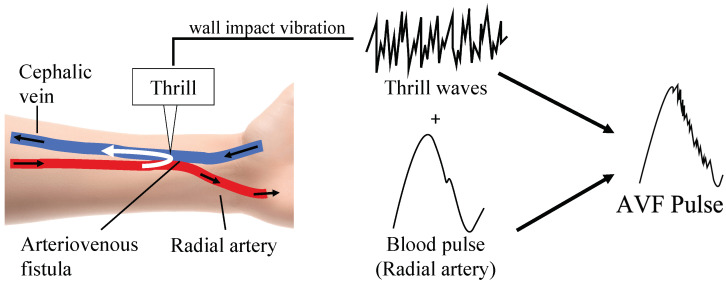
An illustration of the AVF waveforms formed from the pulse and Thrill.

Arteries are composed of a three-layered membrane, with a well-developed tunica media containing several smooth muscles and elastic fibers, making the vessel walls thick and highly elastic. Veins, on the other hand, have immature tunica media and, therefore, have thin and less elastic vessel walls [26]. As blood flows from the artery to the vein via the AVF, the arterial blood that spurts out of the narrow anastomosis hits violently against the thin venous wall, stretching it as it pulsates, as shown in Figure 1. The slight oscillations produced during this process are known as ‘Thrill’ [27]. The waveform of the AVF is observed as a composite of arterial and Thrill waveforms. The Thrill is also used to identify the site of stenosis in clinical practice. When the anastomosis narrows, the blood flow decreases, causing the Thrill to decrease and the pulsation to decrease. However, when the downstream region (body side) narrows, the Thrill decreases and the pulsation increases [28].

### 2.2. Non-Contact Imaging of the AVF

**Figure 2:**
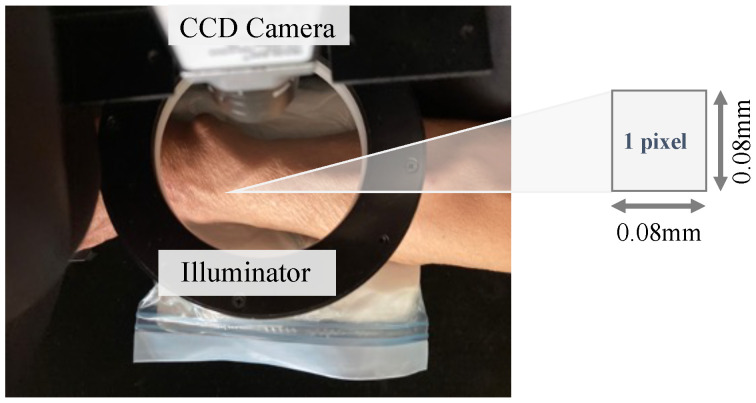
Principle of non-contact imaging.

The arm is positioned so that the AVF is at the centre of the ring-shaped light source, as shown in Figure 2. The color of the light source is the same as in [24], which has shown the effectiveness of blue light. The slight displacement of the AVF due to pulsation and Thrill are captured using a CCD camera as changes in luminance, which are acquired at 40 fps. The saved file format is TIFF, a lossless compression method, and the resulting image file contains AVF waveform data for every pixel (0.08 mm × 0.08 mm). Thus, we obtain the original image data f(i,j) at a two-dimensional location ${\left[i,j\right]}^{T}$.

### 2.3. Moving-Average Filter

**Figure 3:**
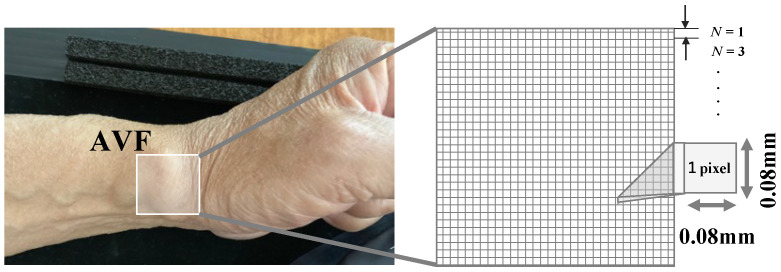
AVF imaging area and the kernel size (N×M) of the moving-average filter.

As non-contact imaging contains a lot of noise from halation and diffuse reflection on the skin, a moving average filter [29] was used to remove this. As shown in Figure 3, this is a general method of smoothing high-frequency components by weighting an N×M matrix called a kernel. In the moving-average filter method, the kernel is defined as an N×M matrix whose component represents a weight coefficient h(m,n). The original image data f(i,j) are convolutionally integrated with the kernel while moving one pixel at a time in a raster scan. Thus, we obtain the moving-averaged value g(i,j), which is expressed with the following equation,

(1) $$g\left(i,j\right)=\sum_{n=-1}^{N}\sum_{m=-1}^{M}f\left(i+m,j+n\right)h\left(m+1,n+1\right).$$

Note that the kernel in this study performs N×N processing, and henceforth, N×N will be expressed as *N*. In addition, we assume *N* is an odd number. Since the moving-averaged output is measured in the time domain, the frequency spectrum of g(i,j) should be calculated with the Fast Fourier Transformation (FFT). Then, the signal and noise power components can be obtained in each specific frequency range. We define the ratio of signal power to noise power as SNR (signal-to-noise power ratio), which can be used for the detection of the AVF stenosis. It should be noted that the signal and noise frequency ranges are discussed in detail in Section 4.

## 3. Preliminary Experiment

To quantify the Thrill waveforms, the frequency bands were identified using the following procedure: The AVF waveform is a composite wave of the radial artery and Thrill, and the frequency characteristics of the radial artery waveform revealed the major harmonic components that form the wave. The frequency characteristics of the AVF waveform were used to determine the difference between the major frequency components of the radial artery and to identify the frequency band of Thrill.

### 3.1. Frequency Response of Arterial Waveforms Using Contact Method

**Figure 4:**
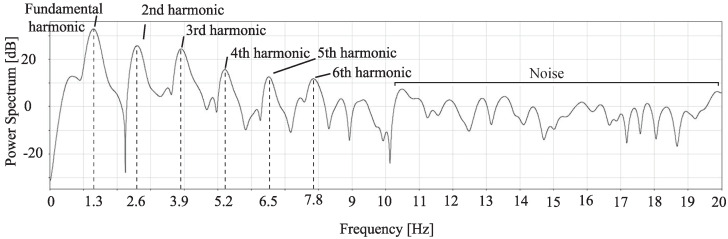
Frequency response of the contact radial artery waveform.

**Figure 5:**
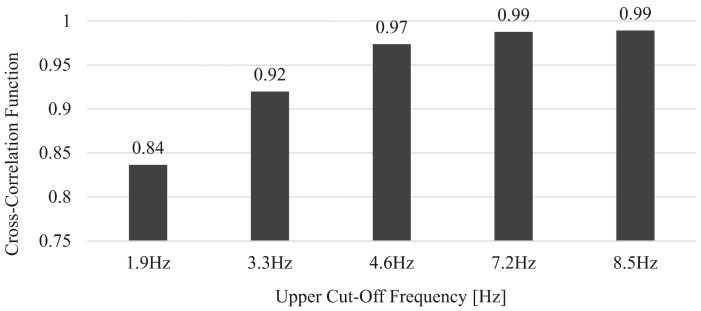
Determination of radial artery waveform components in the contact method by cross-validation.

A pulse wave sensor (BH1792GLC, manufactured by Rohm) was placed in contact with the radial artery and applied with a sampling frequency of 40 Hz for 10 s. The frequency response [30] processed by the band-pass filter (BPF) is shown in Figure 4. As indicated by the lines, the spectral peaks appear in integer multiples (n≤6) from the fundamental to the *n*th harmonic, and the peaks become smaller as *n* increases. It was also assumed to be noise as it was almost flat above the sixth harmonic. The cross-correlation coefficients between the original waveform and each of the waveforms obtained by low-pass filter (LPF) processing with the high cut-off frequency between each harmonic are shown in Figure 5. The cross-correlation coefficient tends to increase continuously and the rate of increase is saturated after 4.6 Hz. It is found that up to the third harmonic are greatly involved in the formation of the waveform, and the contribution of the fourth and subsequent harmonics is small.

### 3.2. Frequency Response of AVF Using Contact Method

**Figure 6:**
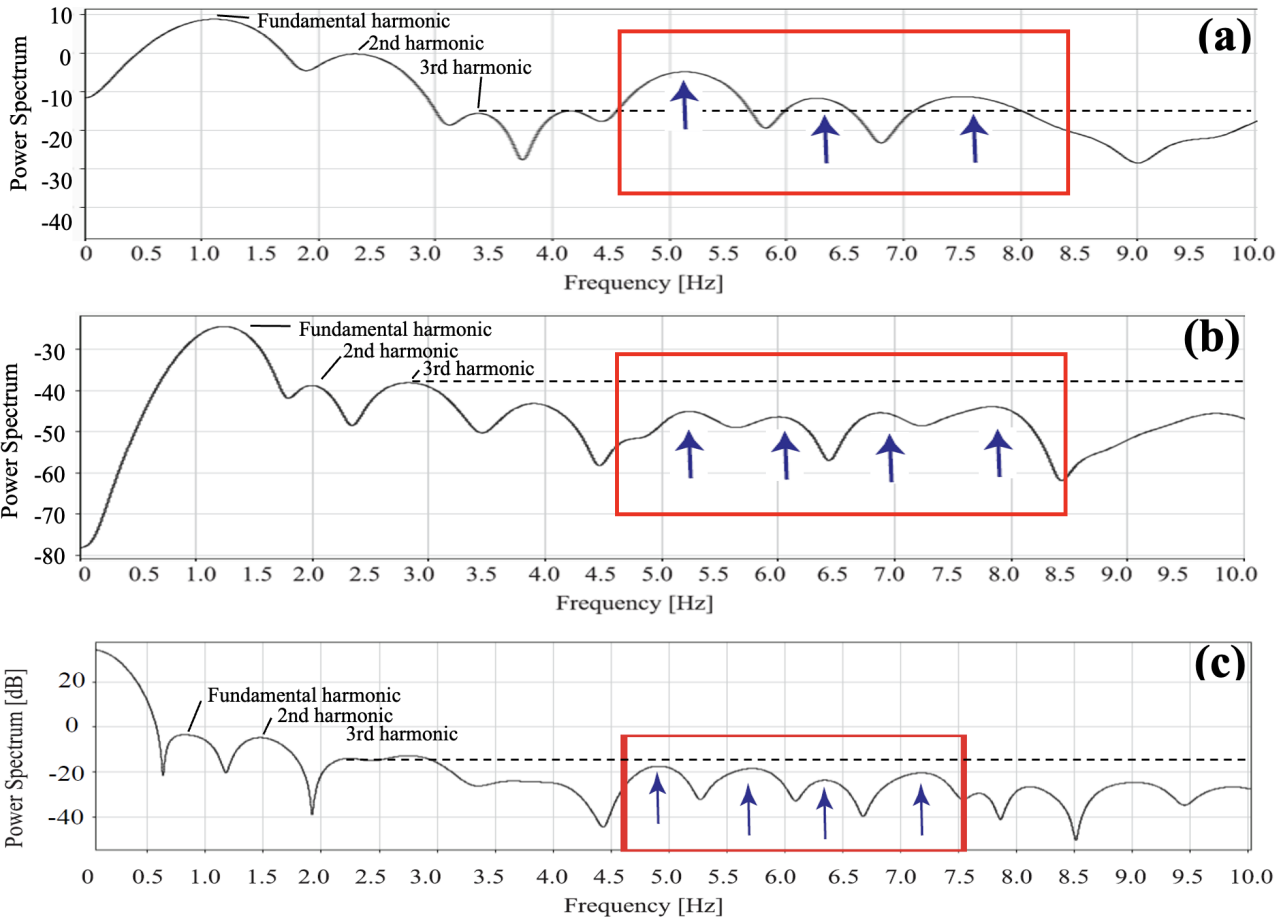
(**a**) Frequency response of the contact AVF waveform, (**b**) frequency response of the non-contact radial artery waveform, and (**c**) frequency response of the non-contact AVF waveform. The blue arrows, the red frames, and the dashed lines indicate the peaks of Thrill components, the estimated frequency range of Thrill components, and the power spectrum level of the 3rd harmonic, respectively.

Figure 6a shows the frequency response of the AVF waveform from the contact method filtered in the same way as Figure 4, with a higher peak than the third harmonic peak in the 5.0 Hz–7.5 Hz band. This phenomenon was not observed in the frequency analysis of the radial artery in either contact or non-contact, as shown in Figure 4 and Figure 6b.

### 3.3. Frequency Response of Arterial Waveforms Using Non-Contact Method

Figure 6b shows the frequency characteristics of the arterial waveforms obtained by the non-contact method through the same filter processing as in Figure 4. Because of the reduced photosensitivity of the non-contact method, the arterial waveforms from the contact method were observed up to the third harmonic, and the peaks after that were nearly of the same power spectrum.

### 3.4. Frequency Response of AVF Using Non-Contact Method

The spectrum of the radial artery decreased as the order *n* increased, but as shown in Figure 6c, the spectrum of the AVF was confirmed to increase in the band around 5–9 Hz. As this phenomenon was not observed in the radial artery, and since this band did not contribute to formation of the waveform, we derived this as the Thrill band, and for this study, we also defined 5–10 Hz as the Thrill frequency band.

## 4. Method

### 4.1. Experiment Procedure

**Figure 7:**
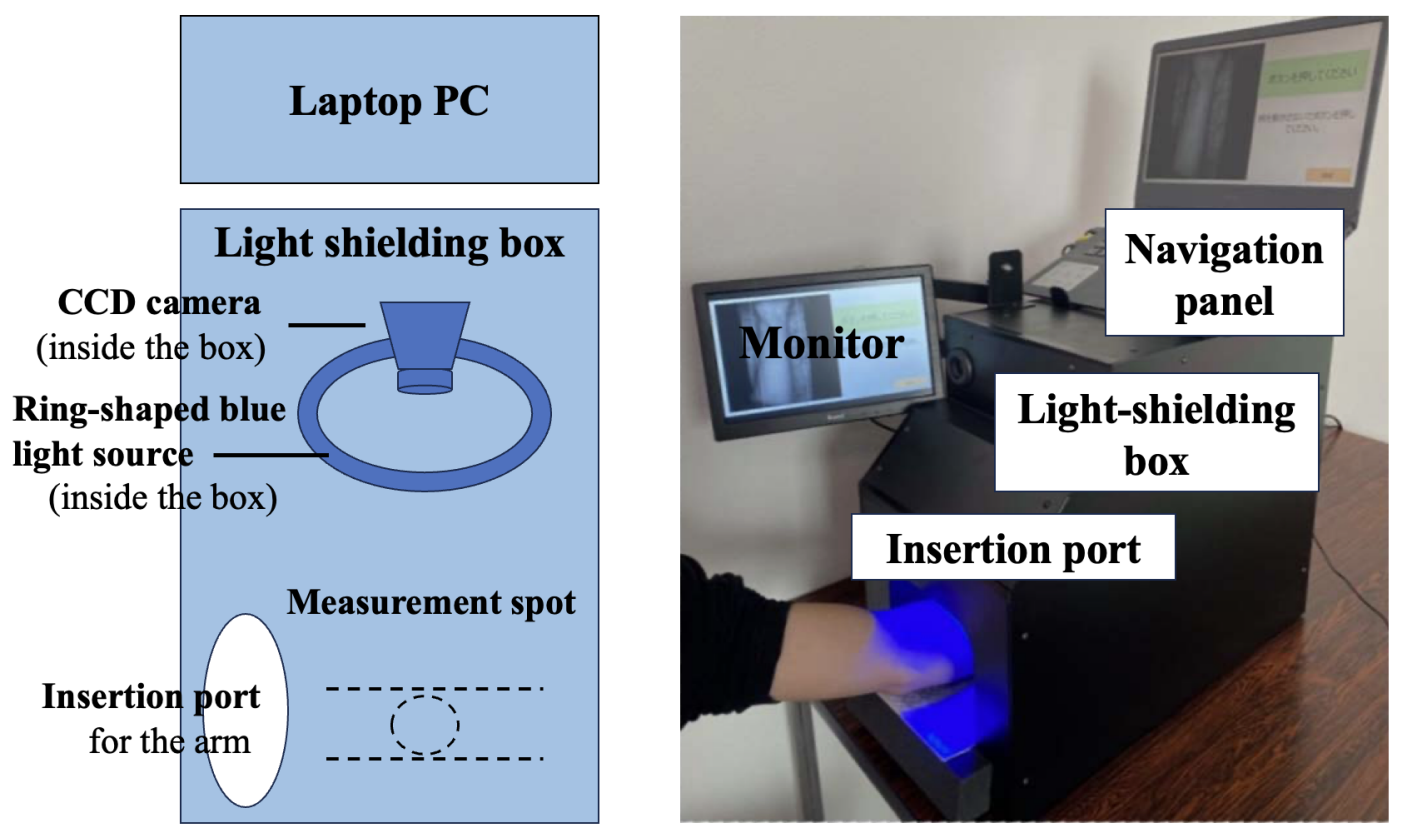
Experimental equipment setup.

As shown in Table 1, there were a total of 206 dialysis data (75 patients, mean age 69.1 years) with normal AVF and 107 dialysis data (17 patients, mean age 70.1 years) with treatable stenosis. In the experiment process the patients were placed in a resting position and their arm was inserted into the insertion port as shown in Figure 7, after which the images were taken for 10 s.

**Figure 8:**
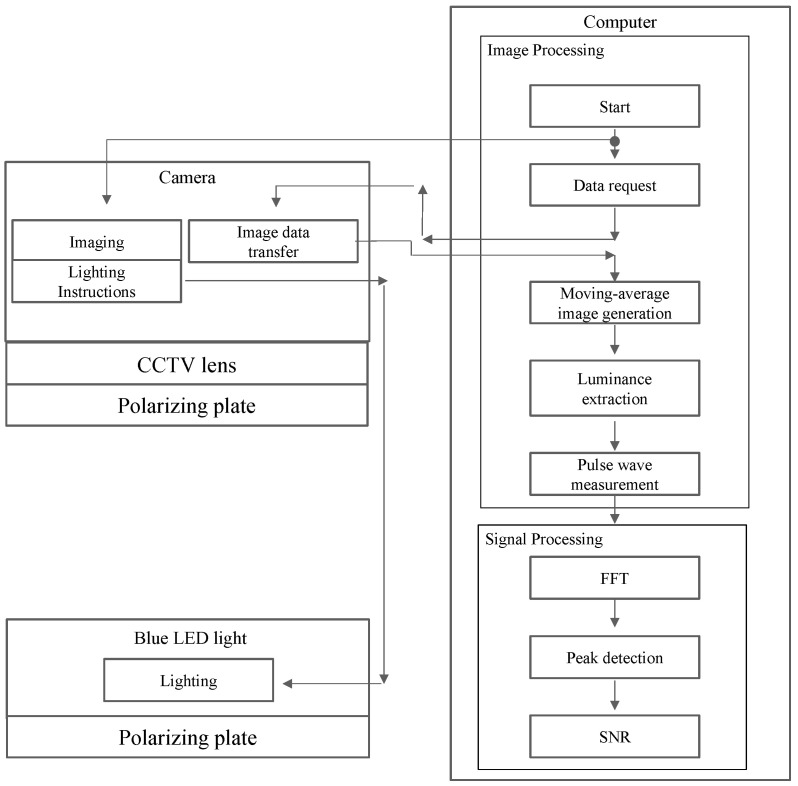
Setup and analysis flow of the non-contact Thrill quantification system.

The setup and processing are shown in Figure 8, where the blue light with an adjusted brightness is captured by the camera as light reflecting from the arm. These received data also contain a lot of halation from common reflection components, which are generated from the midline and protrusions in the arm, as well as having diffuse refraction noise generated from the skin’s surface. Before the camera captures the images, we need to attenuate the halation with a polarizing filter. The data are then captured continuously and converted to TIFF image files. Subsequently, the noise generated by the diffuse reflection is mostly removed by using a moving average method, resulting in a clear AVF waveform. Then, the AVF waveform is converted from the time domain to the frequency domain by using FFT, and the peak values of each spectrum of the Thrill and noise are detected, so that we can then obtain the SNR.

The general criteria for determining AVF stenosis in ultrasound echography are the brachial artery flow volume (FV) ≤ 350 mL/min and the resistance index (RI) ≥ 0.6–0.7 [31,32,33,34], which are the standards of the participating hospitals in this study. So, FV ≤ 350 mL/min and RI ≥ 0.68 subjects who met these criteria were included and classified as the stenosis group in this study. The configuration of this software and hardware was the same [24] as in the previous study. Henceforth, the non-contact imaging method of this study will be referred to as the proposed method.

### 4.2. Quantification

**Figure 9:**
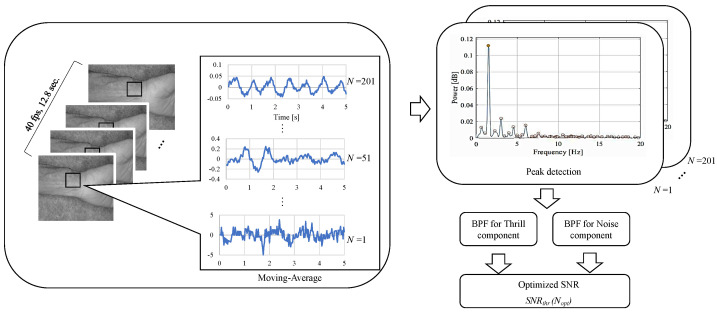
Procedure for $SNR_{thr}\left(N_{opt}\right)$.

As shown in Figure 9, 101 different AVF waves were obtained for one subject by moving the average processing with the kernel size *N* in an odd number ranging from 1 to 201. Next, after extracting the Thrill component with BPF with the low and high cut-off frequencies of 5Hz and 10Hz, respectively, 256 frame size points were processed with a Hanning window and the median of each peak in the power spectrum obtained by FFT calculated to $Peak_{thrill}\left(N\right)$ was obtained. Here, *N* is defined in Equation (1) as the kernel size of the moving-average filter. The $Peak_{noise}\left(N\right)$ is processed in the same way with a BPF (low cut-off frequency 15 Hz, high cut-off frequency 20 Hz) to obtain $Peak_{noise}\left(N\right)$, using the following equation:

(2) $$SNR_{thr}\left(N\right)=\frac{Peak_{thrill}\left(N\right)}{Peak_{noise}\left(N\right)}$$

### 4.3. Determination of $SNR_{thr}\left(N_{opt}\right)$ and Statistical Determination of Normal and Stenotic AVFs

In $SNR_{thr}\left(N\right)$ between the two groups, the normal group and the stenosis group, the optimal kernel $N_{opt}$ is the N that is the largest difference between the two groups, and the optimal SNR is $SNR_{thr}\left(N_{opt}\right)$. After confirming the normality of $SNR_{thr}\left(N_{opt}\right)$ between the normal group and the stenosis group using the Shapiro–Wilk test, the difference between the two groups was statistically evaluated.

## 5. Results

### 5.1. Effects of the Moving Average Process

**Figure 10:**
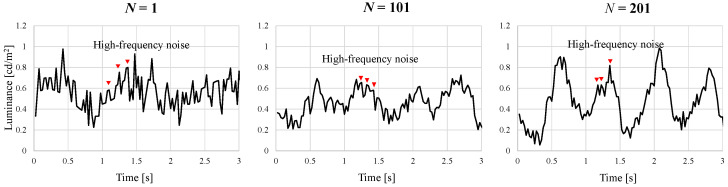
Effects of moving average methods on different kernels.

As shown in Figure 10, at a kernel size of N=1, regular waveforms cannot be visually identified, and the AVF waveform is also indiscernible. High-frequency noise was also observed (indicated by the red triangle markers). At a kernel size of N=101, a slight periodicity became observable. The waveforms at the high-frequency noise did not diminish even with application of the moving average. At a kernel size of N=201, periodicity became clearly evident, and the amplitude increased significantly. The high-frequency noise also did not disappear. Thus, as the kernel size *N* increased, a reduction in high-frequency noise was observed, leading to an increase in the regularity of the AVF waveform. However, the high-frequency noise did not wholly disappear.

### 5.2. Determining $N_{opt}$

**Figure 11:**
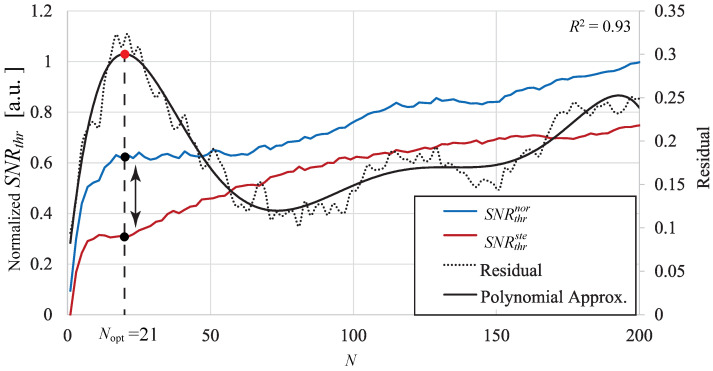
Determining $N_{opt}$.

Figure 11 shows a graph representing the normalized $SNR_{thr}$ for the normal AVF group ($SNR_{thr}^{nor}$), the normalized $SNR_{thr}$ for the stenotic AVF group ($SNR_{thr}^{ste}$), and their residual values, plotted for each kernel *N*. $SNR_{thr}^{nor}$ is represented by the blue line, while $SNR_{thr}^{ste}$ is represented by the red line. The residuals of these SNRs are shown as dashed lines, and the black line represents the smoothed approximation curve of these residuals (6th order polynomial, and coefficient of determination $R^{2}=0.93$). Both $SNR_{thr}^{nor}$ and $SNR_{thr}^{ste}$ exhibited an increasing trend similar to a logarithmic function. The smoothed residual curve peaks at $N_{opt}=21$, which has been determined as the optimal kernel size for quantitatively distinguishing between normal and stenosis conditions.

### 5.3. Quantitative Assessment of Normal and Stenotic Groups

**Figure 12:**
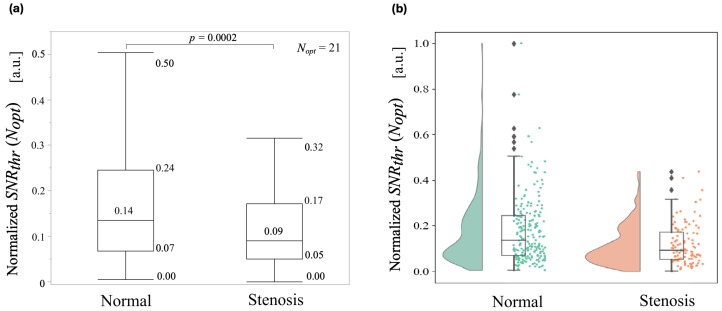
Comparison of normal and stenosis groups by $SNR_{thr}^{opt}$ represented by a box plot in (**a**). Comparison of the distribution of the same group by $SNR_{thr}^{opt}$ represented by a rain cloud plot in (**b**).

Figure 12a,b shows a comparison of $SNR_{thr}^{nor}\left(N_{opt}\right)$ in the normal group and $SNR_{thr}^{ste}\left(N_{opt}\right)$ in the stenosis group. Figure 12a shows the 75th percentile values for the normal and abnormal groups were 0.24 and 0.17, respectively, indicating a higher value for the normal group. The median values were 0.14 and 0.09 for the normal and abnormal groups, respectively, showing a slightly higher value for the normal group. The 25th percentile values were 0.07 and 0.05 for each group, respectively, indicating nearly identical values between the two groups. The Shapiro–Wilk normality test showed that p=0.0001, which did not follow a normal distribution, so a Mann–Whitney U test was performed, and a significant difference of p=0.0002 was observed between the two groups.

The interquartile ranges for the normal and stenosis groups were 0.17 and 0.12, respectively. Figure 12b consists of a probability density, box and dot plots. The probability density shows the data distribution as a smooth shape, while the dot plot shows the plot from Figure 12a with an outlier added into it. The dot plot also shows the frequency distribution. When compared against the stenosis group, the distribution of the normal group is wider from the median and to the higher values, while the stenosis group is concentrated around the median.

## 6. Discussion

### 6.1. Regularity of the Thrill Waveforms

Improvement in waveforms was observed with the increase in kernels, confirming the effectiveness of the moving average method. Although Thrill is said to be random waveforms in clinical practice, the AVF wave showed an increase in the peak value shown in Figure 6c, which was not seen in the frequency response shown in Figure 4 and Figure 6b. This suggests that the Thrill waveforms do not only consist of irregular waves, but also contain regular waveforms.

The arterial wall is subjected to stretching normal to the blood flow and to wall shear stress (WSS) in the tangential direction. Stretching is caused by pulsation and WSS is caused by the spiral laminar flow (SLF) [35,36]. SLF is a widely observed phenomenon in human and animal blood vessels [37] and moderate WSS has been reported to contribute to the suppressive effect of atheroma [38,39,40] and renal damage due to renal artery stenosis [41].

In recent years, the presence of the SLF has also been confirmed in AVFs, and its involvement in vascular maturation has been reported [42,43]. In Marie et al. [44], SLF was observed in 80% of the group capable of sensing Thrill among the 56 subjects with AVF and it was found that Thrill was characterized by spirals rather than turbulence. Their discussion suggests that the frequency band of Thrill waveforms defined in this study may provide evidence of the periodicity created by SLF.

The experimental results regarding the amplitude components of AVF waves from previous studies [24] serve as a quantification method for the stretching of the vessels, which, if demonstrated for the quantification of the WSS component of the Thrill waveforms in this study, would enable the quantification of the two actions of the specific vessel, AVF. However, care should be taken as conditions such as the AVF and blood flow rate differ in Japan compared to other countries, especially the US [2]. It is essential to increase the number of subjects and investigate the regularity and the frequency band of Thrill waveforms defined in this study in the future.

### 6.2. Validity of the Proposed Method

**Figure 13:**
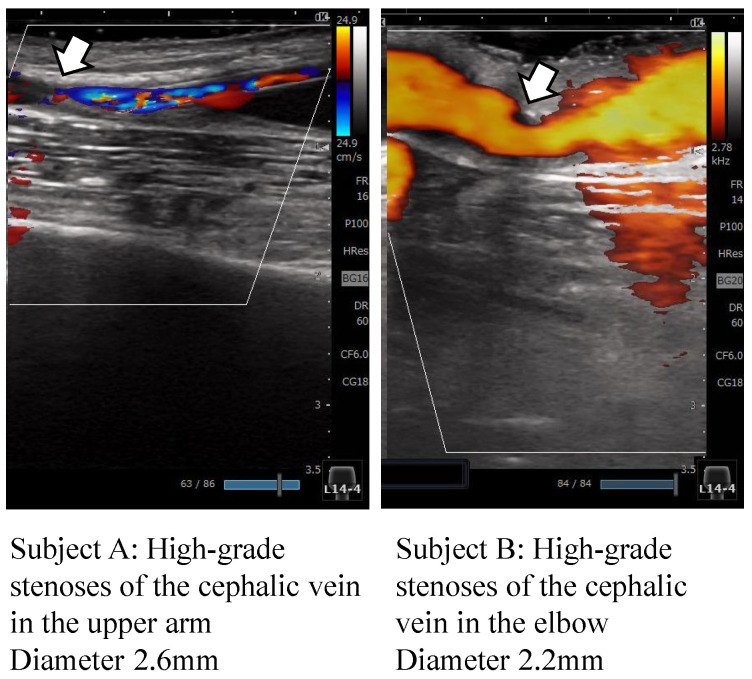
Ultrasound echocardiographic stenosis.

A comparison of $SNR_{thr}^{nor}\left(N_{opt}\right)$ in the normal group and $SNR_{thr}^{ste}\left(N_{opt}\right)$ in the stenosis group, where the frequency band of the Thrill waveforms was 5–10 Hz, showed statistical differences, which were not evident in the box plots. The reason for this may be that the stenosis group in this experiment were subjects who could be treated normally, so the presence or absence of a small amount of stenosis could be shown in the statistics. This paper aimed to identify the frequency components of the Thrill, radial artery and noise. In our investigation, the Thrill component at 5–10 Hz was found to depend on the stenotic changes. Given that the Thrill waveform is sensitive to the blood flow, blood flow variation should have an effect on the stenotic changes, which results in a significant difference in the Thrill component. As shown in Figure 13, to further support the effectiveness of the proposed method, we examined the ultrasound tomographic images of the AVFs of two subjects from the stenosis group and found that subject A had severe stenosis in the brachial vein and subject B had severe stenosis in the radial cutaneous vein at the elbow, indicated by the arrows. When considering the influence of individual differences of the AVF condition on the quantification of stenosis, the experiment was conducted on 92 patients, so the current results include a variety of AVF conditions. Even in these results, the quantification of stenosis changes significantly, so we believe that the effectiveness of quantification of stenosis can be confirmed even with these differences. As future work, we would like to undertake a quantitative study on the quantification of stenosis due to these differences in each individual AVF condition.

### 6.3. Limitation of the Proposed Method

Although we have confirmed the effectiveness of the proposed detection, there are some limitations in the method. For example, stenosis ranges from mild to severe, while also requiring treatment such as PTA. In addition, even with the same degree of stenosis, the measurement results might differ based on the location, for example, if the stenosis is near an anastomosis or on the trunk side. This implies that they might be the reason why it was difficult to distinguish them visually on the graph. We believe that as the collection of stenosis data continues, that in the future the results will become clearer from the evaluation degree and the location of the stenosis. In addition, as this experiment was conducted on patients at a Japanese hospital, the experiment captured changes in luminance, so the results may differ even for the same person due to the degree of shading, sweat, skin color and condition. We plan to investigate how the results are affected in various environments in the future.

## 7. Conclusions

This paper developed reliable stenosis detection based on non-contact AVF imaging, which made use of optical technology. To evaluate the efficiency of the developed detection system, Thrill components were quantified with 92 patients and resulted in 313 data elements, using non-contact imaging in the normal group (206 total data, 75 patients) and in the treatable stenosis group (107 total data, 17 patients). A significant difference was observed in the Mann–Whitney U test between the normal group and the stenosis group, which demonstrates the effectiveness of the developed system.

The ultimate goal of this research is to perform non-contact screening with higher accuracy than auscultation and palpation methods, and to image the stenotic area. Additionally, the non-contact imaging method also allows us to identify the stenosis area and the degree of stenosis by looking at the color changes in the tone, similar to a heat map. This makes it possible to take action even at minor stenosis points. However, as a preliminary study, we cannot offer any additional physiological and pathological reasons for the AVF fluctuations in this study. As we believe that they are an important aspect to study, we will pursue these factors in future work.

Previous research confirmed the effectiveness of a non-contact quantitative method as an alternative to auscultation, and this study showed that it has the potential to be a new alternative method that can eliminate the disadvantages of the auscultation and palpation methods mentioned above. In the future, it would be necessary to further increase the number of subjects and carefully investigate the regularity and the frequency band of the Thrill waveforms defined in this study, taking into account the aforementioned factors.

## Figures and Tables

**Table 1:** Experiment protocol.

Number of patients	92 (normal AVF: 75, AVF with treatable stenosis: 17)
Number of data	313 (normal AVF: 206, AVF with treatable stenosis: 107)
Position	Sitting
Exposure time	10 s
Light source color	Blue
Frame rate	40 fps

## Data Availability

The data presented in this study are only available on request from the corresponding author but with certain limitations to maintain ethics, data privacy and anonymity for the test participants.

## Abbreviations

The following abbreviations are used in this manuscript:

AVF	Arteriovenous fistula
LPF	Low-pass filter
KRT	Kidney replacement therapy
PTA	Percutaneous transluminal angioplasty
QoL	Quality of life
MGD	Multivariate Gaussian distribution
BPF	Band-pass filter
RI	Resistance index
FL	Flow volume
WSS	Wall shear stress
SLF	Spiral laminar flow
FFT	Fast Fourier transform
SNR	Signal-to-noise power ratio
